# Berrywood Asylum

**Published:** 1892-11-19

**Authors:** 


### The Institutional Workshop.

### WITHIN THE ASYLUMS.

[Our Special Commissioner would be glad to visit any public or private asylum if the medical superintendent will communicate with the Editor. This intimation is given owing to several letters which have reached us on the subject.]

### BERRYWOOD ASYLUM.

The Northampton County Lunatic Asylum is situated at Berrywood, about two miles from the station. It occupies an elevated site, commanding a wide view of the town of Northampton, and is sheltered on the one side by the woods from which it takes its name. It constitutes a good specimen of the corridor pavilion type, and of the quadrangular arrangement of the blocks. Were it not for the two-storied fire-proof corridors it might more properly be placed among the pavilion asylums proper. The entrance hall, offices, committee-room, and assistant medical officers' quarters, compose the central block, facing north-west. A short fireproof corridor joins this block to a corridor running at right angles to it; and behind this are the surgery, case-book room, and pathological museum. From either end of the corridor springs another, which passes southwards to the ante-rooms leading to the recreation hall. In the space thus enclosed are the general stores, the kitchen, and its usual adjuncts, the housekeeper's room, and offices. Leading from the ante-rooms on either side is a fireproof passage about 30 feet long, connecting No. 1 Block with the centre. This block contains a long gallery on the ground floor, and another on the first floor. From one end projects a dining-room, at the other end a dormitory, and from the centre a bay. Single rooms are arranged along the other side of the gallery, which is 12 feet wide. The second floor is exclusively for sleeping accommodation. At the end of this block, westward in the case of the women's side, and eastward on the men's, is another fireproof passage, two storeys high. This opens into No. 2 Block, at the south end of which is a large, well-lighted, and airy day-room, and this adjoins a gallery, containing single rooms, passing northwards to a large domitory, which, like the day-room at the other end, has windows in all its walls. This No. 2 Block forms the west side of the women's quadrangle and the east side of the men's. From it a long fireproof corridor begins, and passes to the centre, joining the corridor already spoken of as enclosing the stores and kitchen department. The laundry ward and wash-house are attached to this long corridor on the women's side, and the artisans' ward and workshops on the men's side. The quadrangles are divided into two smaller ones by a dormitory block, which, in its turn, is connected by fireproof passages. These dormitories are recent additions to the asylum, and constitute an important feature of the arrangements. Each block has its own bath-room, and the water-closets are cut off from the wards by ventilated passages. The medical superintendent's residence is attached to the north-east corner of the buildings. The entire accommodation is for 880 patients, and 157 have single rooms.

#### Improvements and Additions.

Since this asylum was built many important additions and improvements have been made. Originally the recreation-hall was used as a central dining-room, where all the patients were brought for meals. In pursuance of the modern endeavour to emphasise the importance of treatment with a view to recovery, the old practice of the central dining-hall has been given up. In its place separate dining-rooms have been erected, so that the patients located in the several blocks may each dine together, and so be freed from the discomforts and drawbacks of the old arrangement. We inspected several of these dining-halls, and found them to be an excellent feature of the institution, well worthy of general imitation. Having regard to the small cost at which patients are maintained in county asylums in this country, it may be well to mention that a close inspection of the diet tables and the food convinced us that both are adequate, and that, in quality, they are quite equal to that supplied by the best hospitals. Dr. Greene, the able medical superintendent, is full of resource, and, like Dr. Clouston, has shown great ingenuity in breaking up the larger day-rooms, so that their appearance, and the comforts which they offer to the patients, are unusually great. Although we should not perhaps altogether agree with the taste he has displayed in the decorations, still no one can fail to be struck with their variety and attractiveness. His selection of pictures, the system of giving each set of dormitories separate quilts of a bright and attractive pattern, and the supply of books everywhere throughout the establishment, such books being freely accessible to all the patients, constitute points for commendation. The fire-drill, instituted by Dr. Greene, is a feature of the establishment, and we could wish that a book by Dr. L. H. Prince, published by Messrs. Blakeley and Co., Chicago, entitled "Fire Protection of Hospitals for the Insane," were better known among asylum superintendents generally than it is at present.

#### New Block for Idiot Children.

This block has been ingeniously planned, and forms one of the pleasantest features at this asylum. It has accommodation for 50 children. The school accommodation, the play-rooms, the single dormitories, the lavatory and bath accommodation are all good. If it is possible to make an idiot child happy, then Berrywood should be an ideal home for these unfortunate little ones. A pavilion has been erected in the grounds, where the children have their meals in the summer time. The playground is cleverly arranged. It contains, among other things, a duck-pond, and two donkeys are kept in constant use for the amusement of the idiots. We wish that the public and asylums' committees, too, could realise the terribly trying character of the duties which nurses and attendants have to discharge in connection with idiot children. Dinner was just over at the time of our visit, and no more favourable opportunity could be selected by anyone who wished to gain an insight into the difficulties and trials of the devoted women who undertake the disagreeable duty of nursing this class of patients. Our humanity compels us to provide accommodation of this character, but the most thoughtful and humane must be the most impressed with a feeling of doubt as to the entire wisdom and justification of taking inordinate pains to prolong the life of incapable, senseless, suffering progeny such as those who find their way into this department of our public asylums.

The Detached Hospital for Infectious Diseases.

The plans of this hospital will be found in Volume 2 of "Hospitals and Asylums of the World," page 37. It is, on the whole, the most complete and best arranged hospital of its kind that we have met with. If it has a fault, we should say that, on the whole, it was over-ventilated. Of course, this is an excellent feature in a climate like our own, but in colder climates, although natural ventilation is mainly relied upon, we fancy such a hospital would be found too fresh for the well-being of most of the patients. Dr. Greene's ingenuity as regards fire-places is well known. His success will be manifest when we state that the largest wards, measuring 40 feet by 22 feet, and 14 feet high, are heated by one fire-place, which has pipes passing through its centre, conveying the cold air from outside into the ward by a grating situated above the mantel-piece. On a cold day this simple contrivance maintains a pleasant temperature throughout the whole ward, and for economy and efficiency we have never met with a stove to beat it. At the present time, as there are no infectious cases in the hospital, it has been utilised for convalescent cases, one ward being made into a large day-room, and the other into a dormitory. This arrangement gives the superintendent a villa residence in the grounds, and the experiment has proved highly beneficial to patients as well as economical and satisfactory. The whole asylum is practically on the open-door system, there being only one ward in each division habitually under lock and key.

The Training of Attendants.

To Berrywood belongs the great credit, thanks first of all to Dr. Greene, and secondly to Dr. Harding (whom we congratulate upon having just secured the M.R.C.P. London), of having made the training at Northampton a real success. Dr. Harding conducts three classes: one for juniors, *i.e.*, attendants in their first year; one for seniors, second year's attendants; and a mixed class devoted to instruction in lunacy. So far it has been the practice to send in the attendants at the end of the first year for the first-aid examination; and at the end of the second for the sick nursing certificate of the St. John Ambulance Association. Having regard to the importance of an adequate training being given to all asylum attendants, we are glad to learn that it is in contemplation to issue a certificate to those attendants at Berrywood, who, after three years' residence, are able to pass such an examination as shall entitle them to receive it. If Dr. Greene is able to carry out this arrangement he will confer a substantial service, not only upon his own staff but upon the staff of all lunatic asylums, who must, soon or later, be then placed in a position to enjoy similar advantages to those offered at Berrywood. It is further desirable that arrangements should be entered into with the County Infirmary, and with other hospitals which train nurses, whereby a nurse may, on paying a fee, obtain a year's training in lunacy at the Northampton County Asylum. In this connection we should like to point out that, as training improves, the standard of the nursing in asylums also improves, and so attracts to the work a higher type of attendants. It is therefore reasonable that the wages given, especially to female attendants, should be considerably increased. It is altogether wrong, in our view, that an asylum committee should give a charge nurse, with a hundred beds, only £25 to £30 per annum. She ought certainly to receive from £30 to £50 per annum, according to length of service and qualifications, as her duties and responsibilities are quite as great, if not greater, than those of the male attendants who receive wages on the higher scale at the present time; and the chance (or at Berrywood the almost certainty) of a pension is as great with the one as the other. At Berrywood a "good service" bonus is given every five years. Many authorities pride themselves upon the fact that the cost of maintaining a patient at a lunatic asylum in the United Kingdom to-day is so small when all the charges are included. We would ask these authorities to reflect whether there is not a minimum charge, and whether it may not be possible, as we believe it to be the fact, that the thirst for economy has tended to reduce the outlay in certain directions below the sum which ought properly to be expended. We believe that one such item is wages, and we are convinced that the well-being of the patients and the just interests of the ratepayers alike demand a reconsideration of the remuneration at present offered, especially to female attendants, who are decidedly underpaid. We cannot conclude this notice without congratulating Dr. Greene upon the excellent staff he has under his charge, and bearing witness at the same time to the remarkable efficiency which characterises all departments of the Northampton County Lunatic Asylum.

